# In-situ formation of Ag nanoparticles in the MAO coating during the processing of cp-Ti

**DOI:** 10.1038/s41598-023-29999-7

**Published:** 2023-02-24

**Authors:** Ł. Maj, Z. Fogarassy, D. Wojtas, A. Jarzębska, F. Muhaffel, A. Sulyok, A. Góral, M. Kulczyk, H. Çimenoğlu, M. Bieda

**Affiliations:** 1 Institute of Metallurgy and Materials Science, Polish Academy of Sciences, Krakow, Poland; 2 Institute for Technical Physics and Materials Science, Centre for Energy Research, Budapest, Hungary; 3 Department of Metallurgical and Materials Engineering, Istanbul Technical University, Istanbul, Turkey; 4 Institute of High Pressure Physics, Polish Academy of Sciences, Warsaw, Poland

**Keywords:** Nanoparticles, Metals and alloys, Characterization and analytical techniques, Transmission electron microscopy

## Abstract

Silver nanoparticle (Ag-NP) containing antibacterial micro-arc oxidation (MAO) coatings have already been synthesized over titanium-based materials via the MAO process employed in silver acetate (AgC_2_H_3_O_2_) containing electrolyte. However, the way of incorporation and in-situ formation of Ag-NPs within the MAO coating have not been documented yet. Present work was initiated to reveal the mechanism of Ag-NP formation within the MAO coatings. Thus, the structure of the MAO coating fabricated on commercial purity titanium in the AgC_2_H_3_O_2_-containing electrolyte was investigated by electron microscopy techniques. To this end, the cross-sectional high-resolution electron microscopy studies were carried out on lamella cut out with the focused ion beam technique, and these investigations were backed by X-ray photoelectron spectroscopy measurements of chemical composition on the surface of the MAO coating. These studies revealed that Ag is dispersed in the form of nanoparticles throughout the coating and that a higher density was confirmed closer to the micro-pores.

## Introduction

Antibacterial surfaces are of high need as current estimates suggest that from 4 to 10% of patients receiving dental implants develop postoperative infections^1^. Bacterial colonization and biofilm formation harm the substratum, where bacterial attachment can lead to implant failure along with inflammations of peri-implant tissues^2^. To reduce this risk, several attempts have been made to develop Ag-containing antibacterial coatings on Ti-based substrates, considering the antibacterial nature of Ag^3–6^. It has been stated that the fraction and size (i.e. surface-area-to-volume ratio) of the Ag particles play an important role in the antibacterial efficiency of coatings. In general, larger surface-area-to-volume ratios allow the substantial release of Ag^+^ ions to interact with the lipopolysaccharide molecules forming the outer membrane of the bacteria, disrupting its integrity, and penetrating deep into the cell, damaging its DNA^7,8^. In this way, Ag affects the functioning of bacteria cells, resulting in the elimination or significant reduction of their activity. However, excessive release of Ag^+^ ions may lead to a cytotoxic effect on the tissues. It was confirmed that blood concentrations of Ag should not exceed 300 ppb to prevent eventual side effects^9^. It has been reported that allowable Ag concentration in the plasma sprayed coating should be in the range of 2 wt.%, which is regarded as the cytotoxic limit for coatings applied to metallic biomedical alloys according to the reports in the literature^10^.

As the most promising surface modification technique for titanium implants, the micro-arc oxidation (MAO) process has the potential for fabricating thick, adherent and micro-porous titanium oxide coatings containing Ag-NPs, whose antibacterial characteristics could be adjusted by altering the concentration of the antibacterial agents in the electrolyte. Recently, a superior combination of bioactivity and antibacterial efficiency against *Staphylococcus aureus* has been obtained from the MAO coatings containing 1.14 and 0.7 wt% Ag for commercially pure titanium (cp-Ti) and Ti-6Al-4V alloy, respectively^11,12^.

In the literature, Ag-NPs-incorporated antibacterial MAO coatings were fabricated by adding Ag-NPs or Ag-containing chemicals/salts (silver acetate and silver nitrate) into the electrolyte. While Ag-containing salt-added electrolytes favour the formation of very fine Ag-NPs (~ 1–3 nm) within the MAO coatings, Ag-NP-added electrolytes tend to produce MAO coatings containing relatively larger Ag-NPs (approximately tens of nm^13^). When the size of the Ag-NPs is of concern, the addition of Ag-containing salt-added electrolytes tends to favour the formation of MAO coatings exhibiting more efficient antibacterial properties. Aydogan et al.^11^ reported that even such small addition of the AgC_2_H_3_O_2_ to the base electrolyte as 0.001 mol/l forms MAO coating having ~ 99.98% antibacterial efficiency against *S. aureus* after 24 h of incubation. Furthermore, Ag concentration in simulated body fluid was kept at levels below the cytotoxic limits for the human body, that is, 1.5 ppm after 14 days of the immersion^12^. Although studies showed that antibacterial MAO coatings produced with the addition of Ag-containing salts are more effective against bacteria than standalone NPs, the reason behind this (participation of Ag in the MAO coating in the form of NPs) remains unknown. Moreover, too little attention has been regarded to detailed microstructure studies aimed at explaining the way of Ag is incorporated from the AgC_2_H_3_O_2_ into the MAO coating.

Thus, within this work a detailed microstructural analysis, with particular attention on high resolution electron microscopy, was carried out in order to find the explanation of the very high antibacterial effectivity of MAO coatings having *in-situ* formed Ag-NPs produced with AgC_2_H_3_O_2_ presented in^11^. In this study, electron microscopy observations were supported by surface-sensitive X-ray photoemission spectroscopy (XPS) analysis, often applied in efficient study of Ag-NPs, also for the TiO_2_-Ag system^14–19^. The results of the present study allowed to propose mechanisms of Ag-NPs formation from used salts and provide new insights into the fabrication of antibacterial coatings by simple and cost-effective approach.

## Materials and methods

In this work, titanium of commercial purity (grade 4) was used as a substrate material. The initial material, purchased from Wolften company in the form of a rod having a diameter of 50 mm, was subjected to a plastic deformation covering multi-pass hydrostatic extrusion reducing its diameter down to 5 mm. Details of this processing were described elsewhere^20^. The rod after hydrostatic extrusion was cut into cylinders of 4 mm in height, ground with sandpapers and ultrasonically cleaned in ethanol and distilled water to remove the surface contamination before the MAO processing, aimed at fabrication of the coating.

Micro-arc oxidation of titanium substrates was performed in a stainless steel vessel filled with the electrolyte containing 0.1 M of Na_2_HPO_4_ and 0.002 M of AgC_2_H_3_O_2_ inorganic salts, dissolved in distilled water. The samples were immersed in this electrolyte throughout the process and alternating pulses of the positive and negative electric potentials of 300 and 60 V, respectively, were applied to them using a bipolar pulsed power supply for an overall time of 5 min. Each electric pulse was maintained for 0.6 ms, separated by 0.4 ms of breaks between them. The temperature of the process was kept at ~ 22 °C.

Phase analysis of the produced coatings in macroscale was performed using the grazing incidence X-ray diffraction (GI-XRD) technique at an angle of 3° with a Bruker D8 Discover diffractometer equipped with a Co anode. Phase composition was achieved by indexing of the recorded spectrum (with a 2θ = 0.01° angular step and an acquisition time of 0.25 s per step) with the help of the DIFFRACplus software and the PDF4 crystallographic database. The crystallite size was evaluated by the analysis of the broadening of the peaks on the XRD spectrum using the Williamson–Hall (W–H) method performed in the HighScore Plus computer program.

The SEM micro-scale observations of the coatings as well as the preparation of the lamellae for TEM studies were done using a ThermoFisher Scios 2 Dual Beam microscope, equipped with an EasyLift nanomanipulator for lift-out of the samples (FIB method). The TEM nanoscale microstructure observations were carried out with a ThermoFisher Themis G2 200 kV FEG microscope equipped with a ThermoFisher Ceta™ bottom camera, a Fischione HAADF/STEM detector and a ChemiSTEM™ energy dispersive X-ray spectrometer (EDS).

The chemical composition of the coatings was determined by the XPS method. X-rays from a water-cooled Al anode (providing 20 mA emission current using 15 keV excitation) irradiated the sample from an 18 mm work distance and spectra were detected using a special retarding field cylindrical mirror analyzer type DESA 150 (Staib Instruments Ltd, Germany). Spectra were recorded with a constant 1.5 eV energy resolution at 0.1 eV energy steps. The measurements were performed under an ultra-high vacuum of 1 × 10^–9^ mbar. The sample surface was cleaned to remove contamination before the XPS measurement through 30 min of ion sputtering with an Ar^+^ ion beam using 1 keV energy, set to a glancing angle of 15°. To determine the peak intensities, the Gaussian/Lorentzian peak model function was fitted to the measured peaks involving the Shirley-type background subtraction. Additionally, concentration calculation took place assuming the homogeneous distribution model and using sensitivity factors from^21^.

## Results and discussion

**Figure 1:**
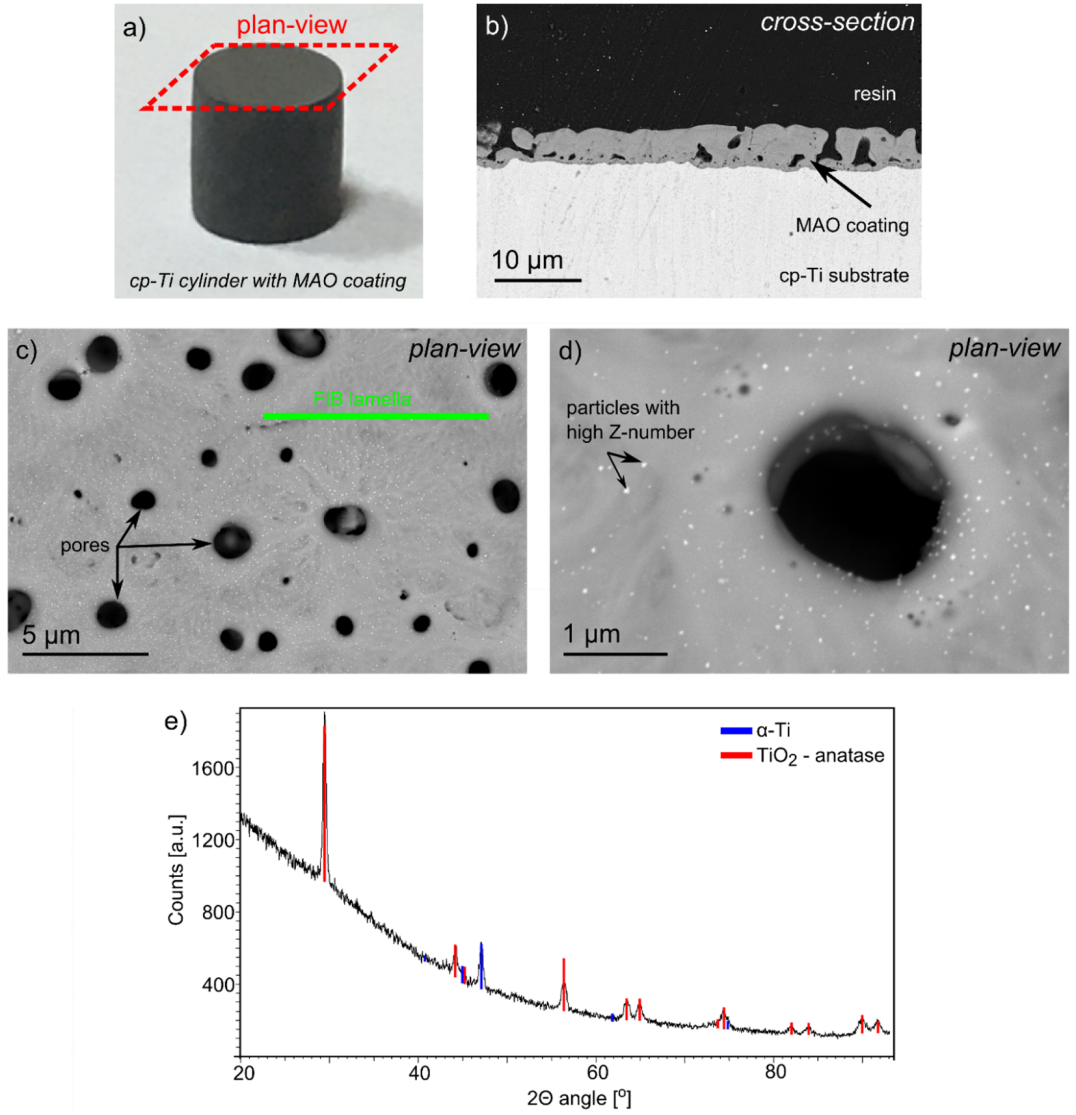
Macroscale photo of the cp-Ti sample with MAO coating (**a**), cross-section SEM/BSE image (**b**), plan-view SEM/BSE image of MAO coatings with the addition of Ag (**c**,**d**) and GI**-**XRD pattern recorded from the top surface of the MAO coating (**e**).

The SEM/BSE images presenting the MAO coatings with the addition of Ag are shown in Fig. 1. They show that the whole surface is covered with the coating material with an average thickness of ~ 5 µm (Fig. 1b). The MAO coating exhibits a high porosity with numerous micro-pores of diameters ranging from several tens of nm to several µm (Fig. 1c). The images obtained at higher magnifications indicate the presence of a large number of fine spherical particles of high-atomic-number-elements that cover almost homogenously the whole coating surface (Fig. 1d). It was observed that some of these particles protrude from the surface of the coating. The FIB lamella was cut out from the place shown in Fig. 1c in a way to present the cross-section of the coating while avoiding larger porosity sections. SEM observations showed that the surface of the coating is free from larger agglomerates of particles, confirming that the MAO processing conditions were optimised efficiently. Additionally, indexing of the GI-XRD diffraction pattern revealed the presence of the anatase phase of the TiO_2_ with an average crystallite size of ~ 40 nm (Fig. 1e). Furthermore, a weak signal from the substrate (α-Ti) was recorded. The lack of signal from the Ag in the GI-XRD pattern is explained by an insufficient signal-to-noise ratio, similar to the observation of Aydogan et al.^11^.

**Figure 2:**
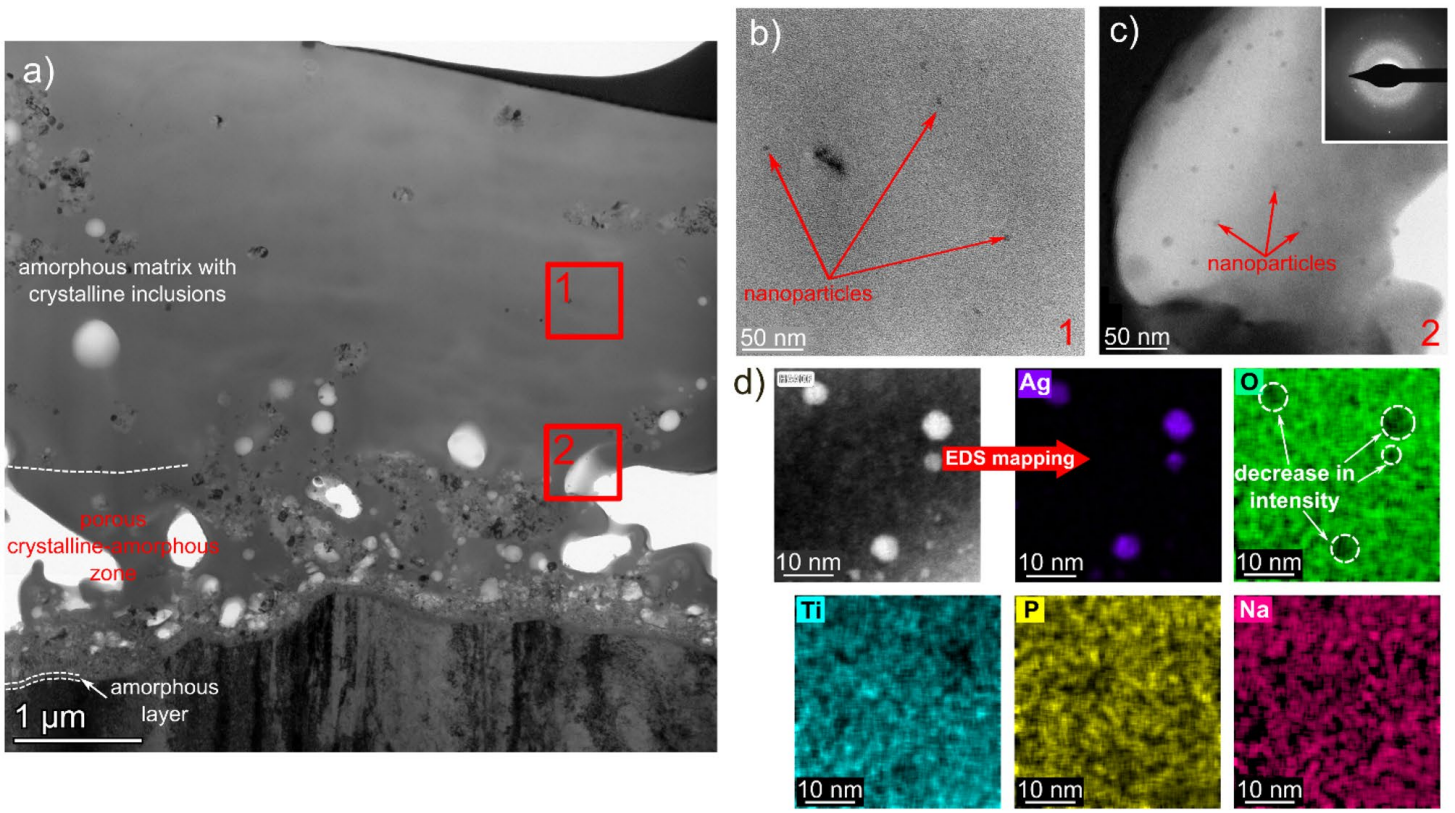
TEM/STEM images of MAO coating with Ag addition: BF images presenting a general overview of the coating (**a**), Ag-NPs in the central part of the coating (**b**), and agglomerates near the pores (**c**) as well as STEM/HAADF image showing Ag-NPs and accompanying EDS maps presenting distribution of Ag, O, Ti, P, and Na distribution (**d**).

The TEM/BF images obtained from the coating cross-section showed the microstructure features typical for the MAO-coated samples (Fig. 2). Presence of a thin continuous amorphous layer (up to 50 nm in thickness) can be noticed at the coating/substrate interface, similar to the observations in^20^ securing a good bonding between them (Fig. 2a). Above this, a porous zone consisting of a mixture of crystalline and amorphous oxides was formed. An almost fully amorphous zone (with only small crystalline inclusions) was visible farther from the substrate. The formation of amorphous zones can be attributed to a high cooling rate, which is up to 10^8^ K/s due to very short micro-arc durations (mostly less than 1 ms). On the other hand, the observations carried out at higher magnifications revealed the presence of fine spherical particles with a size < 10 nm dispersed in the amorphous matrix (Fig. 2b,c). These particles are non-uniformly distributed within the amorphous matrix: a much higher amount was located close to the pores than for the other areas of the coating. Only a small number of NPs has been observed in the areas far from the pores. The EDS maps of chemical composition presented in Fig. 2d confirmed that the investigated particles are characterised by the increased signal of Ag with a simultaneous decrease in the number of counts from the other elements: Ti, O, Na, and P (dashed circles). This is an indicator that the Ag-NPs almost entirely aggregate within the amorphous phase, as confirmed by the SAED pattern presented as an inset of Fig. 2c. Moreover, Fig. 2c also shows the typical halo for amorphous phases, together with numerous spots of intensity originating from the crystalline Ag. The previous observation coincides with the studies carried out by Mosquera et al.^22^ that showed the addition of Ag tends to inhibit the amorphous-to-anatase transformation for the pulsed cathodic arc-deposited Ag-TiO_2_ coatings. In addition, our recent microstructure studies of MAO-fabricated TiO_2_ coatings obtained with Na_2_HPO_4_ electrolyte proved the formation of mostly amorphous transformation with a small contribution of the anatase phase residing mainly in the areas near the interface^20^.

**Figure 3:**
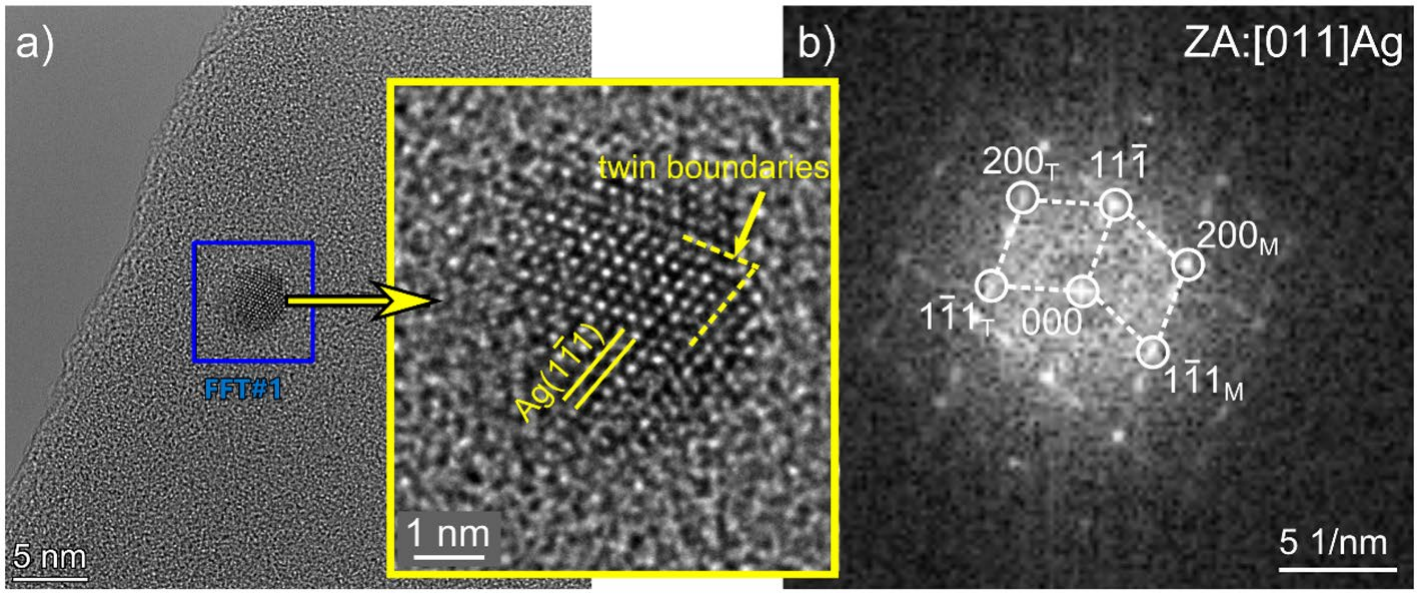
HREM image of single Ag nanoparticle oriented along [011] direction magnified in inset (**a**) and accompanying FFT (**b**) with reflexes from the matrix (M) and twins (T).

The phase analysis of the NPs was performed based on the HREM images and indexing of the accompanying Fast Fourier Transform patterns (FFT) showed that they are of α-Ag (Fig. 3a,b). In addition, the planar defects typical for Ag (twins) are also observed, which are the consequence of its low stacking fault energy^23^. The twins are represented by additional bright spots visible in the FFT image. Described results of a complementary approach covering the plan-view and the cross-sectional (on FIB lamellae) high-resolution electron microscopy studies show direct evidence of the nm-sized metallic Ag formation and its distribution in the titanium oxide-based coating during the MAO process. Despite the different studies made regarding the MAO process, few attempts have been made to investigate the way Ag incorporates into the coating from the AgC_2_H_3_O_2_ electrolyte during the MAO processing. Aydogan et al.^12^ confirmed by SEM observations that Ag-based particles agglomerate on the surface of the MAO samples were present, mainly in the surroundings of the micro-pores and craters. On the other hand, Wang et al.^24^ carried out the TEM study of the hydrothermally treated MAO coatings on the TiO_2_ pieces scratched off the surfaces. Although the presence of only one random Ag nanoparticle was shown, the distribution of the Ag-NPs and the way in which they embed into the coatings were elucidated.

**Figure 4:**
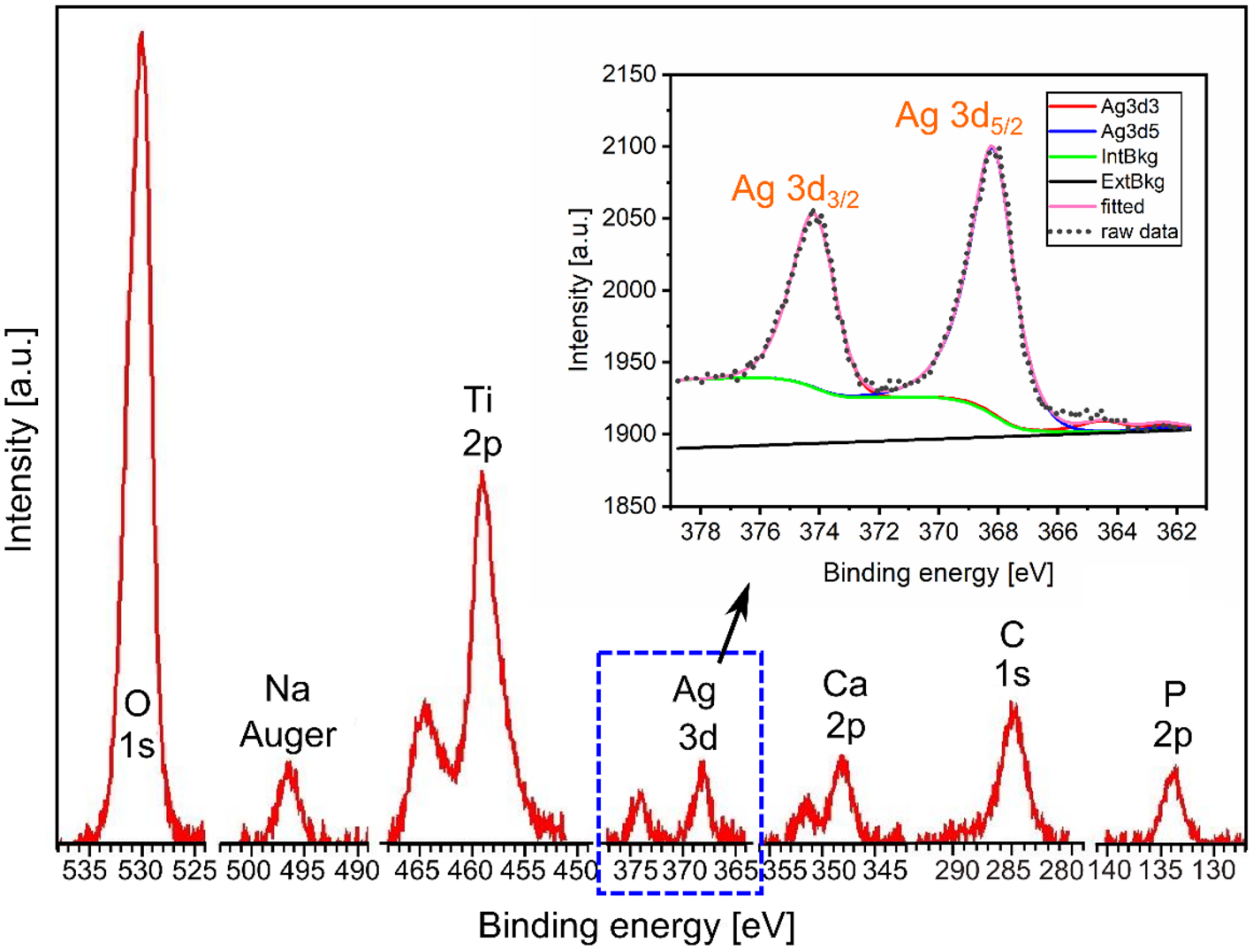
XPS spectrum presenting peaks (without carbon) obtained from the surface of MAO coating (spectrum of the Ag-3d level shown as inset).

The XPS spectra acquired from the MAO coating produced with the electrolyte containing a mixture of Na_2_HPO_4_ and AgC_2_H_3_O_2_ proved successful embedding of Ag in its top surface (detected signal comes from a depth less than 10 nm). In addition, a small amount of calcium was also observed. Separate XPS measurements were carried out to reveal the exact shape of the Ag-3d peaks and determine its chemical state. With a long detection time, it provided a good signal-to-noise ratio despite the low intensity of the Ag signal (inset of Fig. 4). The binding energy of Ag-3d_5/2_ was determined using a value of 284.6 eV for an adventitious carbon used as a reference peak and found to be 368.2 ± 0.2 eV. It is identical to the nominal value of the binding energy provided by the metallic Ag. Furthermore, based on the quantitative analysis of the XPS spectra, one may conclude that up to 0.5 at% of Ag was confirmed on the surface (Table 1).

**Table 1:** Results of the quantitative analysis of the XPS spectra.

Ti [at.%]	O [at.%]	P [at.%]	Na [at.%]	Ca [at.%]	Ag [at.%]
19.4	67.7	4.9	5.5	2.0	0.5

**Figure 5:**
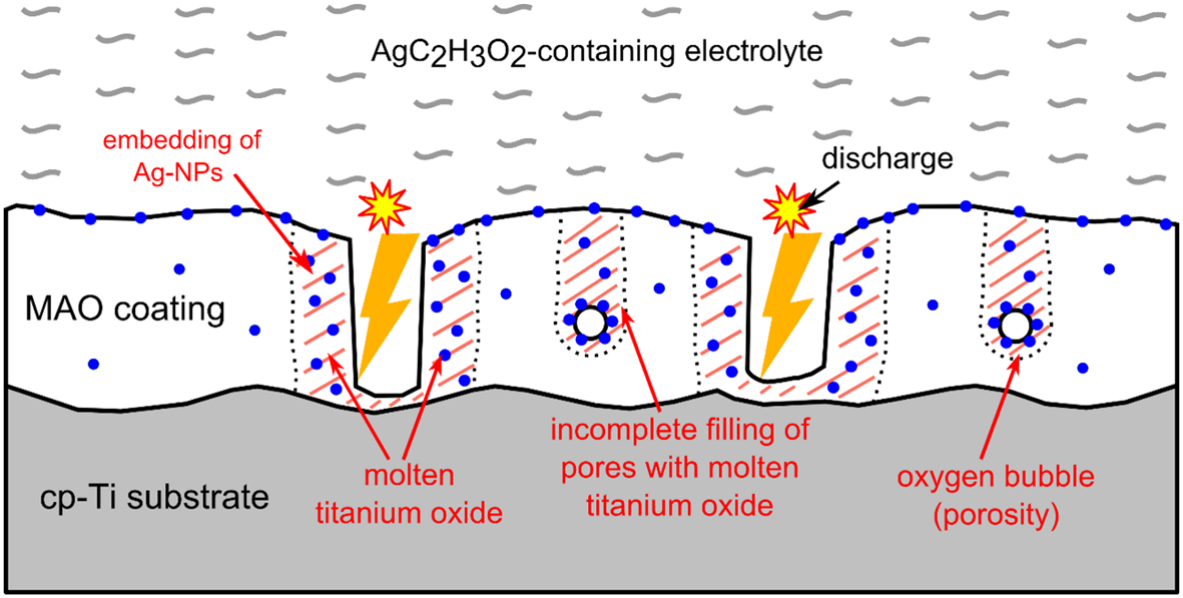
Scheme presenting the mechanism of incorporation of Ag-NPs in titanium oxide coating from AgC_2_H_3_O_2_ containing electrolyte.

Based on the results presented above, a model is proposed describing the formation of the coating and the incorporation of Ag-NPs from the AgC_2_H_3_O_2_-containing electrolyte (Fig. 5). Firstly, a large number of e^-^ is generated due to the oxidation of titanium that yields to OH^-^ ions arising from the decomposition of H_2_O in the cathode. The presence of OH^-^ and Ti^4+^ produces Ti(OH)_4_ and then TiO_2_. The solubility of AgC_2_H_3_O_2_ in distilled water at 20 °C is 10.2 g/l, thus, its amount of 0.002 M should decompose according to the following reaction:

1 $${\text{AgC}}_{2} {\text{H}}_{3} {\text{O}}_{2} \left( {\text{s}} \right) \to {\text{Ag}}^{ + } \left( {{\text{aq}}} \right) + {\text{CH}}_{3} {\text{COO}}^{ - } \left( {{\text{aq}}} \right)$$

The electric field applied between the anode and cathode forces the Ag^+^ ions, present in the electrolyte, towards the sites of the coating growth, and according to Clyne et al.^25^, this is the volume of the TiO_2_ coating formed near the centre of the discharge channel that melts during a single discharge event. In the meantime, Ag^+^ ions are prone to oxidation (in the vicinity of O^2-^ ions) forming Ag_2_O oxide which, in turn, decomposes at temperatures exceeding 300 °C into Ag atoms and O_2_ molecules^26^. The decomposition of Ag_2_O occurs due to a rapid rise in temperature in the discharge area up to even 12,000 K (estimated by optical spectroscopy) and a strong temperature gradient associated with it^25^. As a result of the mixing of Ag decomposed from AgC_2_H_3_O_2_ delivered to the discharge vicinity and molten TiO_2_, Ag embeds into the growing TiO_2_-based coating. Mosquera et al.^22^ showed that Ag does not dissolve in TiO_2_, but it is pushed away, forming Ag-NPs and this explains the presence of the Ag-NPs in the areas near the cavities, substantially, as well as near the upper surface of the produced coating. In addition, it enables the production of the Ag-NPs having a much smaller size compared with the conventional Ag-NPs provided by manufacturers (typically several tens of nm).

## Conclusions

The application of high-resolution electron microscopy for investigation of MAO coatings prepared on the surface of cp-Ti with AgC_2_H_3_O_2_-containing electrolyte helped to describe its microstructure in detail and henceforth to prove that:

- The application of electrolytes containing Na_2_HPO_4_ and AgC_2_H_3_O_2_ promoted the formation of a porous amorphous-crystalline mixed titanium oxide coating with small metallic Ag-NPs (sized < 10 nm) embedded within the coating.
- The density of the Ag-NPs was found to be much higher in the vicinity of the micro-pores and craters; moreover, some of the NPs are even protruding from the upper surface of the coating.
- The proposed mechanism of incorporation of Ag-NPs from the AgC_2_H_3_O_2_ into the MAO-produced coating comes from the decomposition of the AgC_2_H_3_O_2_ in the electrolyte, delivery of Ag^+^ ions into the discharge site after application of electric field, and subsequent precipitation of the NPs during the growth of the coating.

In summary, this work revealed the mechanism of MAO coating formation when AgC_2_H_3_O_2_-containing electrolytes were used intended for future biomedical applications i.e. to cover the titanium-based implants with antibacterial coating. The in-situ formation of Ag-NPs close to the surface and porosity of the coating explains their high antibacterial effect, because in this way the Ag-NPs may directly influence the DNA of the bacteria attached to the coating. The use of AgC_2_H_3_O_2_ in MAO is highly competitive with the addition of Ag-NPs supplied since it is less costly and more effective in terms of antibacterial effect without jeopardising the biocompatibility.

## Data Availability

The datasets used and/or analysed during the current study is available from the corresponding author on reasonable request.
